# Dietary Supplementation with the Red Seaweed *Porphyra umbilicalis* Protects against DNA Damage and Pre-Malignant Dysplastic Skin Lesions in HPV-Transgenic Mice

**DOI:** 10.3390/md17110615

**Published:** 2019-10-29

**Authors:** Susana Santos, Tiago Ferreira, José Almeida, Maria J. Pires, Aura Colaço, Sílvia Lemos, Rui M. Gil da Costa, Rui Medeiros, Margarida M. S. M. Bastos, Maria J. Neuparth, Helena Abreu, Rui Pereira, Mário Pacheco, Isabel Gaivão, Eduardo Rosa, Paula A. Oliveira

**Affiliations:** 1 Department of Veterinary Sciences, University of Trás-os-Montes and Alto Douro (UTAD), 5001-801 Vila Real, Portugal; suusanacoelhosantos@gmail.com (S.S.); tiagoterras55@gmail.com (T.F.); josecfralmeida@gmail.com (J.A.); joaomp@utad.pt (M.J.P.); acolaco@utad.pt (A.C.); silviaalexandralemos@gmail.com (S.L.); 2 Centre for the Research and Technology of Agro-Environmental and Biological Sciences (CITAB), 5001-801 Vila Real, Portugal; rmcosta@fe.up.pt (R.M.G.d.C.); erosa@utad.pt (E.R.); 3 Animal and Veterinary Research Center (CECAV), 5001-801 Vila Real, Portugal; 4 LEPABE—Laboratory for Process Engineering, Environment, Biotechnology and Energy, Faculty of Engineering, University of Porto, Rua Dr. Roberto Frias, 4200-465 Porto, Portugal; mbastos@fe.up.pt; 5 Molecular Oncology and Viral Pathology Group, IPO-Porto Research Center (CI-IPOP), Portuguese Institute of Oncology of Porto (IPO-Porto), 4200-072 Porto, Portugal; ruimmms@gmail.com; 6 Faculty of Medicine, University of Porto (FMUP), 4200-450 Porto, Portugal; 7 CEBIMED, Faculty of Health Sciences, Fernando Pessoa University, 4200-150 Porto, Portugal; 8 LPCC Research Department, Portuguese League against Cancer (NRNorte), 4200-172 Porto, Portugal; 9 Research Center in Physical Activity, Health and Leisure (CIAFEL), Faculty of Sports, University of Porto, 4200-450 Porto, Portugal; mneuparth@hotmail.com; 10 ALGAplus, Lda., PCI-Creative Science Park, 3830-352 Ílhavo, Portugal; helena.abreu@algaplus.pt (H.A.); rui.pereira@algaplus.pt (R.P.); 11 Department of Biology and CESAM, University of Aveiro, 3810-193 Aveiro, Portugal; mpacheco@ua.pt; 12 Department of Genetic and Biotechnology, CECAV, UTAD, 5001-801 Vila Real, Portugal; igaivao@utad.pt; 13 Department of Agronomy, UTAD, 5001-801 Vila Real, Portugal

**Keywords:** K14HPV16, genotoxicity assay, papillomavirus, cancer

## Abstract

Some diet profiles are associated with the risk of developing cancer; however, some nutrients show protective effects. *Porphyra umbilicalis* is widely consumed, having a balanced nutritional profile; however, its potential for cancer chemoprevention still needs comprehensive studies. In this study, we incorporated *P. umbilicalis* into the diet of mice transgenic for the human papillomavirus type 16 (HPV16), which spontaneously develop pre-malignant and malignant lesions, and determined whether this seaweed was able to block lesion development. Forty-four 20-week-old HPV^+/−^ and HPV^−/−^ mice were fed either a base diet or a diet supplemented with 10% seaweed. At the end of the study, skin samples were examined to classify HPV16-induced lesions. The liver was also screened for potential toxic effects of the seaweed. Blood was used to study toxicological parameters and to perform comet and micronucleus genotoxicity tests. *P. umbilicalis* significantly reduced the incidence of pre-malignant dysplastic lesions, completely abrogating them in the chest skin. These results suggest that *P. umbilicalis* dietary supplementation has the potential to block the development of pre-malignant skin lesions and indicate its antigenotoxic activity against HPV-induced DNA damage. Further studies are needed to establish the seaweed as a functional food and clarify the mechanisms whereby this seaweed blocks multistep carcinogenesis induced by HPV.

## 1. Introduction

Seaweeds are an important nutritional resource in many parts of the world and have various health-promoting biological activities [1,2]. Frequent consumption of seaweeds in South-East Asian countries has been associated with low incidence rates of chronic diseases, such as cancer [3,4]. Seaweeds contain high amounts of vitamins, fibers, and minerals, potentially contributing to a balanced diet if consumed regularly [5]. They are also a source of bioactive compounds with antioxidant, antitumor, anti-inflammatory and antiviral bioactivities [1,6], which make seaweeds popular functional foods for disease prevention [7,8]. In fact, some seaweeds contain natural compounds with significant pharmacological potential for cancer prevention and treatment [9,10]. *Porphyra umbilicalis* (Bangiophyceae) is an intertidal red seaweed (Rhodophyta), and its genome has already been disclosed, contributing to clarify the evolution of red seaweeds [11]. *P. umbilicalis* is used as food and is particularly appreciated for its unusually high protein content, vitamins, and fibers [12]. In a per-portion comparison, 100 g of wet weight of *P. umbilicalis* contains more total fiber (3.8 g) than apples and bananas, 2.0 g and 3.1 g, respectively. Regarding the presence of vitamins, 8 g of *P. umbilicalis* contains 9 mg of vitamin C [5]. Among other uses, *P. umbilicalis* was found to improve the nutritional profile of meat preparations, increasing its antioxidant properties [13]. Cofrades et al. [12] demonstrated that this seaweed presents significant benefits to human health, being itself a functional food. However, to our knowledge, the potential of *P. umbilicalis* in the prevention of cancer has never been evaluated.

Many cancers are associated with infection by oncogenic viruses, most commonly, human papillomavirus (HPV) [14]. High-risk HPVs are responsible for 630,000 new cancer cases per year, mainly cervical cancer but also other anogenital cancers and some oropharyngeal carcinomas [15]. The most common high-risk HPVs are HPV16 and 18, associated with 73% of HPV-related cancer cases [15]. Compounds from seaweeds, like carrageenan from Rhodophyta, showed remarkable preventive effects against HPV infection [16,17]. Recently, *Fucus vesiculosus* (Ochrophyta) showed significant in vitro activity against HPV-positive oropharyngeal cancer [18]. The present study addresses, for the first time, the potential of *P. umbilicalis* as a functional food to block the development of HPV-induced pre-malignant dysplastic lesions through its incorporation into the diet of HPV16-transgenic (K14HPV16) mice [19]. These animals develop multi-step cutaneous lesions induced by the HPV16 oncogenes, from hyperplastic foci through dysplastic patches to invasive squamous cell carcinomas, and may be used to test chemopreventive strategies [20,21]. K14HPV16 mice also show a debilitating syndrome, characterized by systemic inflammation, stunted growth, and chronic hepatitis, which progresses to overt cachexia with age [22]. We took advantage of these features to confirm whether *P. umbilicalis* could help countering this syndrome or would actually raise any safety issues by aggravating any toxicological parameters.

## 2. Results

### 2.1. General Findings 

At the end of the experiment, all animal survived, and none showed signs of distress. There were no significant differences in body weight between groups at any time point, and food intake was similar throughout the experiment (data not shown). Higher water consumption was observed in groups II and IV of transgenic animals (7.80 g ± 1.43 and 7.06 g ± 0.79, respectively), compared to groups I and III of wild-type animals (5.11 g ± 1.14 and 3.61 g ± 0.46, respectively). Thus, significant differences were not observed among groups (*p* > 0.05). The relative weight of internal organs is reported in Table 1. There was a statistically significant decrease (*p* = 0.016) in the relative weight of lungs between group I and group III.

### 2.2. HPV-Induced Lesions and Hepatic Histology

**Figure 1:**
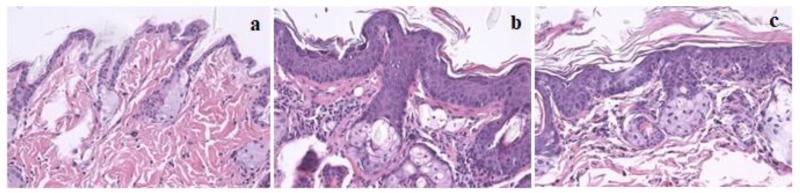
Skin histology samples of female FVB/n mice, magnification 200×, hematoxylin and eosin (H&E) staining: (**a**) Normal skin histology in wild-type groups (I and III); (**b**) Epidermal hyperplasia in K14human papillomavirus(HPV)16 transgenic mice; (**c**) Epidermal dysplasia in K14HPV16 transgenic mice.

Histological analysis of skin chest and ear samples are reported in Table 2. *P. umbilicalis*-supplemented-diet group II showed epidermal hyperplasia in 100% of the mice, while base diet-fed group IV only showed 36.4% of epidermal hyperplasia. Therefore, there were statistically significant differences concerning the skin chest among supplemented and not supplemented animals (*p* = 0.004). On the other hand, the *P. umbilicalis*-supplemented-diet group II mice did not show epidermal dysplasia, while 63.6% of non-supplemented mice showed epidermal dysplasia. Regarding ear samples results, there were no statistically significant differences between the supplemented and the non-supplemented groups. Figure 1 shows skin histological samples for (a) normal skin, (b) epidermal hyperplasia, and (c) epidermal dysplasia. On histological analysis, all mice showed normal hepatic morphology (data not shown). 

### 2.3. Serum Biochemical Parameters

The serum biochemical parameters are registered in Table 3. There were no significant differences between *P. umbilicalis*-supplemented-diet and base diet-fed groups. 

### 2.4. Comet Assay

**Figure 2:**
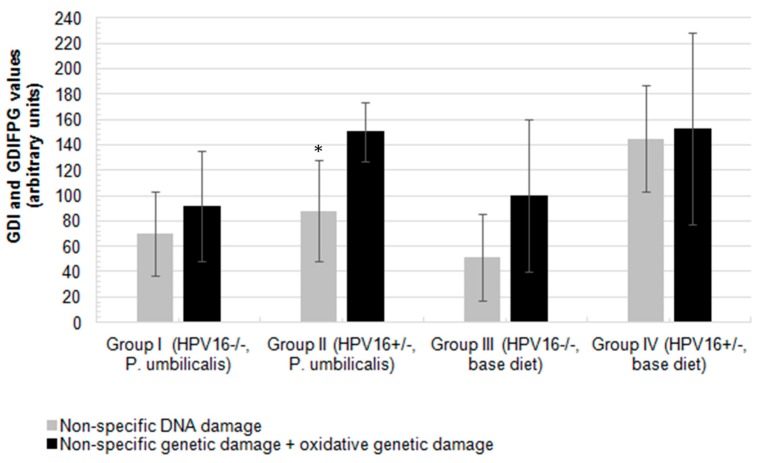
Mean values of non-specific genetic damage index (GDI, grey) and oxidative genetic damage index resulted from the assay with an extra step of digestion with formamidopyrimidine DNA glycosilase (FPG, GDI_FPG_, black), measured in peripheral blood cells of female FVB/n mice (*n* = 11 for each group). Asterisk (‘*’) represents statistically significant differences (*p* = 0.006) relative to non-specific DNA damage (grey) in group II, in comparison to the control group IV (grey). Bars represent the standard deviation.

**Figure 3:**
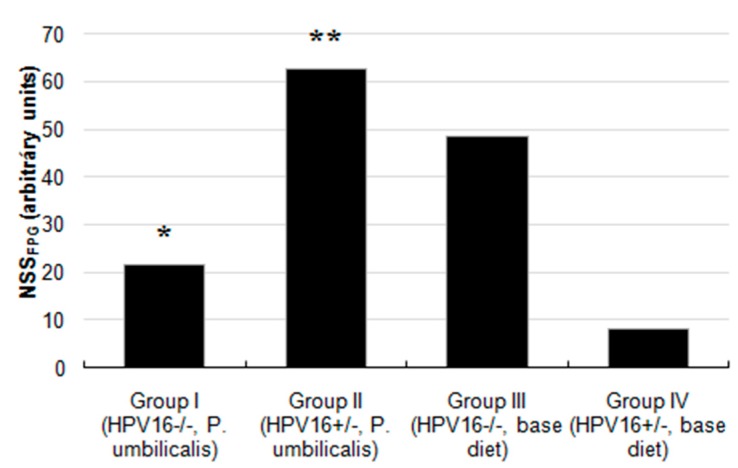
Values of net FPG-sensitive sites from the comet assay with an additional FPG step to detect oxidized purine bases. ‘*’ represents statistically significant difference (*p* = 0.001) in group I, in relation to group III; ’**’ represents statistically significant difference (*p* < 0.001) in group II, in relation to group IV (*n* = 11 for each group).

The *P. umbilicalis*-supplemented-diet transgenic group II showed a significantly lower basal genetic damage index (GDI) compared with the base diet-fed group IV (*p* = 0.006). There were no statistically significant differences concerning formamidopyrimidine DNA glycosylase (FPG) incubation (GDI_FPG_) among groups (Figure 2), but the *P. umbilicalis*-supplemented-diet group I displayed significantly (*p* = 0.001) lower net enzyme-sensitive sites (NSS_FPG_), compared with the base diet-fed group III (Figure 3). Higher (*p* ˂ 0.001) NSS_FPG_ levels were detected in *P. umbilicalis*-supplemented-diet transgenic group II, compared with group IV (base diet). 

### 2.5. Micronucleus Test

**Figure 4:**
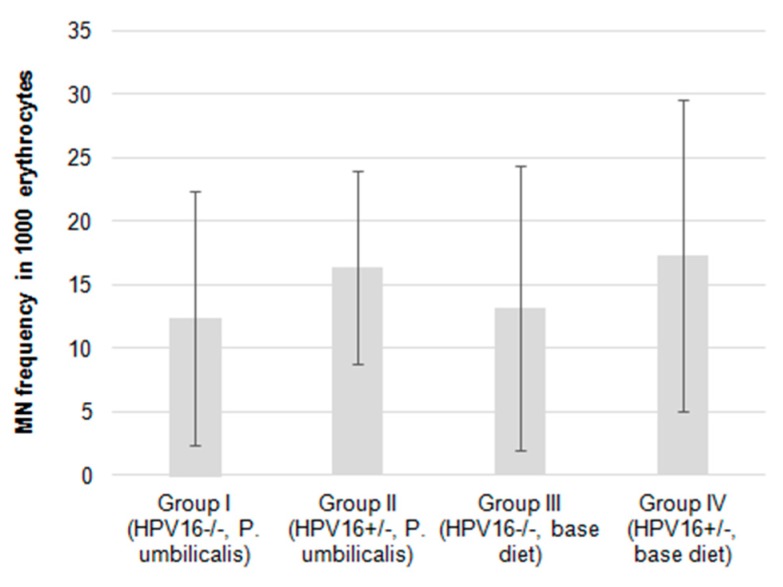
Mean frequency of micronuclei in 1000 erythrocytes of female mice FVB/*n* during the experimental study (*n* = 22 for each group). Bars represent the standard deviation.

The micronucleus (MN) frequencies were slightly lower in the *P. umbilicalis*-supplemented-diet group I compared with the base diet-fed group III. The same applies to the *P. umbilicalis*-supplemented-diet group II that showed a lower MN frequency compared to the base diet-fed group IV (Figure 4), although these differences did not reach statistical significance.

## 3. Discussion

Cancers induced by high-risk HPVs remain among the most frequent and deadly, especially in developing countries, where the implementation of vaccination and screening programs have been less successful [15]. In fact, effective prevention efforts are critical to reduce the incidence of many types of cancer, either by withdrawing risk factors (e.g., oncogenic viruses and tobacco toxins) or by screening and treating pre-malignant lesions. A growing body of data suggests that some foods and nutrients also contribute to preventing the development of cancer [23]. Seaweeds are recognized as an important component of balanced diets in many world regions, and some species have shown anticancer activity. In general, addition of *P. umbilicalis* to food increases the concentration of some minerals (K, Ca, Mg, Mn, and Fe), amino acids, and consequently total protein [12]. Also, *P. umbilicalis* is a rich source of polyunsaturated fatty acids which are precursor of various cell components, allowing to maintain cellular homeostasis [13,24]. However, *P. umbilicalis*, a widely consumed red seaweed, has not yet been studied for its chemopreventive activity against cancer. 

Here, we report the first data showing that *P. umbilicalis* is able to block the development of pre-malignant lesions in a mouse model of HPV16-induced cancer. In K14HPV16 transgenic mice, the *cytokeratin 14* (*K14*) gene promoter/enhancer is used to specifically target the expression of all the HPV16 early region genes, including the key drivers of malignant transformation E6 and E7, to epithelial basal cells in keratinized squamous epithelia [25]. The HPV16 E6 and E7 oncoproteins induce the degradation of the cellular p53 and retinoblastoma (pRb) proteins, allowing unchecked proliferation, survival, and accumulation of genetic mutations and driving carcinogenesis [26,27]. In consequence, this animal model develops multi-step lesions of the skin [21]. Our group and others have characterized these lesions and shown that their development may be blocked pharmacologically, using investigational and commercial drugs [20,22,28]. HPV16 causes cancers in mucosal surfaces such as the cervix rather than the skin, but skin lesions in this model closely follow the same histological and molecular pattern of multi-step carcinogenesis observed in cervical cancer [29,30]. In the present study, *P. umbilicalis* dietary supplementation completely abrogated the progression of hyperplastic epidermal lesions to the dysplastic stage in chest skin. Thus, we observed an increase of hyperplastic epidermal lesions from 36.4 to 100% in transgenic mice supplemented with *P. umbilicalis* in comparison with the control group. This suggests that *P. umbilicalis* was effective in preventing the progression of HPV16-induced lesions in this animal model. In the ear skin, this effect was less dramatic: supplementation with *P. umbilicalis* resulted in a decrease the incidence of dysplasia from 63.6 to 22.2%. Previous studies have shown that lesions in the chest and the ear progress along similar pre-malignant epidermal hyperplasia and dysplasia to form invasive squamous cell carcinomas (SCCs) and that SCCs originating from the chest skin are poorly differentiated and tend to metastasize to regional lymph nodes, while SCCs from the ear skin are well differentiated and only invade locally [19]. It is interesting to notice that *P. umbilicalis* preferentially inhibited the development of the most aggressive type of lesions from the chest, rather than that of the well-differentiated SCCs arising from the ear skin. The present study employed a short-term approach for *P. umbilicalis* supplementation, and additional studies are needed to confirm its long-term effects. While it is possible that long-term supplementation may enhance the protective effects of this seaweed, it is also possible that these effects may wane over time. *P. umbilicalis*, being a rich source of polyphenols, has a broad range of biological activities including anti-inflammatory, immune-modulatory, antioxidant, cardiovascular protective, and anti-cancer actions [13,31]. Polyphenols show a beneficial influence on skin aging and dermal diseases (cancer) [32]. A combination of vitamins A, C, and E with natural extracts obtained from *P. umbilicalis* can show significant effects in skin protection against UV radiation, preventing DNA damage and inflammation, and can also act on cell renewal [33]. Inflammation is associated with the development and malignant progression of most cancers [34]. Therefore, its control has a negative impact on tumor development. As we previously reported, K14HPV16 mice showed increased numbers of tumor-associated leukocytes compared with wild-type animals [35]. In fact, multiple studies have shown that the development of lesions in these animals depends on tumor-associated inflammation and that the administration of anti-inflammatory drugs is able to block tumor progression [22,28,30,35,36,37]. Our results support the hypothesis that dietary supplementation with *P. umbilicalis* may have chemopreventive effects against pre-malignant lesions induced by HPV16 and associated with inflammation. Another interesting possibility is that *P. umbilicalis* might be able to reduce the activity of the *cytokeratin 14* gene promoter, thereby decreasing the expression of the HPV16 transgenes and slowing down the development of lesions. Elucidating the mechanisms underlying this chemoprevention should be the focus of future studies on this red seaweed. We also wished to confirm that dietary supplementation with *P. umbilicalis* was safe, especially in the presence of a chronic debilitating condition. The present results do not show any influence of *P. umbilicalis* over animals’ behavior or the clinico-physiological variables analyzed, such as food intake and body weight. There were also no changes in the intermediate and hepatic metabolism associated with *P. umbilicalis*, at both the biochemical and the histological level. This is particularly interesting, as this model was previously shown to develop chronic hepatitis easily, as part of a wasting syndrome characterized by chronic systemic inflammation, presumably in response to the lesions induced by HPV16 transgenes [22]. The reduced relative mass of the lung in animals treated with *P. umbilicalis* of the group I is likely due to the presence of different amounts of residual blood following cardiac puncture. Two genotoxicity assays were used to study possible mutagenic risks posed by *P. umbilicalis*, i.e., the comet assay, also known as single-cell gel electrophoresis (SCGE), and the MN test [38]. The use of both assays allows the gathering of complementary information, as DNA damage detected by the comet assay occurs earlier, is rather short-lived, and does not require cell division to become evident. On the contrary, the MN test detects DNA-strand breaks that are not repaired and persist beyond mitosis, giving rise to clastogenic lesions or to aneuploidy [39,40,41]. *P. umbilicalis* reduced non-specific GDI in HPV16-transgenic mice. Relatively to the oxidative damage revealed by FPG treatment, there was a side effect consisting in the stimulation of the antioxidant system. It has been shown that some foods with antioxidant action induce a slight increase in reactive oxygen species (ROS) to activate the antioxidant system and thus strengthen the defenses against stronger exogenous genotoxic stimuli [42]. Concerning wild-type mice, the seaweed had no effect over the non-specific GDI but significantly reduced oxidative DNA damage. Importantly, the MN test did not reveal any significant differences between groups, although there was a trend for HPV16-transgenic mice to show slightly higher MN frequencies. Overall, these results suggest that *P. umbilicalis* does not induce any genotoxicity. The seaweed may even have DNA protective effects in specific contexts, and the results observed in wild-type and HPV16-transgenic animals deserve further study. Currently, there is only very limited knowledge concerning the DNA protective or damaging effects of seaweeds, and some recent studies in *Drosophila melanogaster* suggested that some seaweed species may provide protection against different genotoxic insults [43].

## 4. Material and Methods

### 4.1. Animals

K14HPV16 transgenic mice were generously donated by Drs. Jeffrey Arbeit and Douglas Hanahan from the University of California, through the National Cancer Institute Mouse Repository (USA). In these animals, expression of the whole early region of HPV16 is controlled and directed to basal epithelial cells by the *cytokeratin 14* gene promoter [29]. Forty-four female mice on an FVB/n background (22 transgenic HPV16^+/−^ mice and 22 wild-type HPV16^−/−^ mice) at 20 weeks of age were used. By 20 weeks of age, these animals start undergoing a critical change in their lesions, which progress from a purely hyperplastic stage to a more advanced, dysplastic stage [29,30]. Preventing this transition theoretically blocks further progression towards malignancy, so we chose to act within this time frame. The animals were genotyped as previously described [25]. This experimental assay was approved by the University of Trás-os-Montes and Alto Douro Ethics committee (approval no. 10/2013) and the Portuguese Veterinary Authorities (approval no. 0421/000/000/2014). 

### 4.2. Diet Preparation

*Porphyra umbilicalis* was harvested from Mindelo beach (41°18′36.8′’N 8°44′25.9′’W), Vila do Conde, Portugal (October 2015). This seaweed was taken to the ALGAplus company, Ílhavo, Portugal, where it was dried for 24 h in a controlled-temperature chamber (25 °C), to 10–12% humidity. Then, the seaweed was milled and incorporated at 10% (w/w) into a standard mouse diet (Diet Standard 4RF21, Ultragene, Italy). The chosen concentration (10%) was based on research performed with *D. melanogaster* [44]. The seaweed and the base diet were finely milled, mixed, and granulated to form new pellets (2 mm in diameter), using an industrial mixer and adding 6.67% (v/w) of water. The base diet for the control groups was milled and granulated without including *P. umbilicalis*. The newly made pellets were dried at 40 °C during 48 h and stored at 4 °C until used.

### 4.3. Experimental Conditions

The animals were maintained in accordance with the Portuguese (Portaria 1005/92 dated October the 23rd) and European (EU Directive 2010/63/EU) legislation, under controlled conditions of temperature (23 ± 2 °C), light–dark cycle (12 h light/12 h dark), and relative humidity (50 ± 10%). The animals were identified individually and housed in hard polycarbonate cages (Eurostandard Tipo IV 1354G, Tecniplast, Italy; Eurostandard Tipo IV S 1500U, Tecniplast, Italy) using corncob bedding (Ultragene, Santa Comba Dão, Portugal) and environmental enrichment with paper rolls. Water and food access were provided ad libitum.

### 4.4. Experimental Design

The animals were divided into four groups: group I (HPV16^−/−^, *n* = 11) and group II (HPV16^+/−.^, *n* = 11) received *P. umbilicalis*-supplemented diet, while group III (HPV16^−/−^, *n* = 11) and group IV (HPV16^+/−^, *n* = 11) received the base diet, during 22 consecutive days. Animal body weight, body condition, behavior, mental status, grooming, ears and whiskers, mucosae, posture, respiratory and cardiac frequency, hydration status, answer to external stimuli, and feces were monitored weekly, along with water and food consumption. At the end of the experiment, all animals were sacrificed by xylazine–ketamine overdose followed by cardiac puncture exsanguination, according to FELASA guidelines [45]. A complete necropsy of the animals was performed, and the internal organs (liver, right and left kidney, spleen, lung, heart, bladder, and thymus) were collected. Skin samples (chest and ear) and internal organs (liver) were collected for histological analysis. 

### 4.5. Histological Analysis

The left ear was collected and longitudinally sectioned for histological analysis. Chest skin was harvested from the lower cervical to the diaphragmatic zone, forming a square of approximately 1 cm^2^. Skin samples from the chest and ear and liver samples were fixed in 10% neutral buffered formalin and processed for hematoxylin and eosin (H&E) staining to classify HPV-induced cutaneous lesions and any toxic hepatic lesions attributable to *P. umbilicalis*. Skin samples were classified as normal, epidermal hyperplasia, or epidermal dysplasia. Normal epidermis was characterized by the presence of only 1 or 2 cellular layers and a keratin layer, while hyperplastic and dysplastic lesions showed over 3 cellular layers. Additionally, dysplastic lesions showed marked nuclear pleomorphism and suprabasal mitotic figures. Liver samples were classified as normal liver, grade I hepatitis, grade II hepatitis, and grade III hepatitis, as previously described [22].

### 4.6. Biochemical Analysis of Serum

Blood samples were collected by cardiac puncture and centrifuged at 1400× *g* for 15 min to isolate serum. The concentration of glucose, albumin, and total protein, as well as alanine aminotransferase (ALT), aspartate aminotransferase (AST), and gamma glutamyltransferase (GGT) were determined through spectrophotometric methods using an autoanalyzer (Prestige 24i, Cormay PZ), in order to detect potential metabolic disorders and hepatotoxic effects. 

### 4.7. Genotoxicity Assays 

#### 4.7.1. Comet Assay

The alkaline comet assay (pH > 13) was performed as previously described [46,47]. Briefly, slides were precoated with normal-melting-point (NMP) agarose. For each animal, 4 slides were prepared (2 for performing the assay with the repair enzyme and the other 2 for the assay without the enzyme). Approximately 10 µL of blood was diluted in 200 µL of ice-cold phosphate-buffered saline (PBS) in a 0.5 mL microtube to prepare a cell suspension. Twenty μL of cell suspension was mixed with 70 μL of 1% low-melting-point (LMP) agarose, and 8 drops were placed onto the 4 precoated slides (2 replicates per slide). The slides were immersed in a lysis solution and rinsed three times. In order to specifically measure oxidatively damaged DNA, namely, 8-oxoguanines and other altered purines, 2 slides were incubated for 30 min with formamidopyrimidine DNA glycosylase (FPG), a DNA lesion-specific repair enzyme which converts oxidized purines into DNA single-strand breaks, donated by Professor Andrew Collins (University of Oslo, Norway). Slides with and without FPG treatment were gently immersed in a freshly prepared alkaline electrophoresis solution to allow DNA unwinding. Subsequently, the cells were electrophoresed in the same solution for 30 min at 25 V and a current of 300 mA. Following electrophoresis, the cells were immersed in PBS followed by distilled water, dehydrated in 70% and absolute ethanol, and air-dried. For visual scoring, DNA was stained with 1 μg/mL of 4,6-diamidino-2-phenylindole (DAPI) solution (Sigma-Aldrich Chemical Company, Spain) and visualized using a fluorescent microscope (OLYMPUS R XC10, U-RFL-T). The relative fluorescence intensity of head and tail (extent of DNA migration) was used as an indicator of DNA damage. One hundred comets (50 comets per gel) were evaluated to obtain a GDI in a scale ranging between 0 and 400 arbitrary units. Scores for GDI_FPG_ were subtracted from those for GDI to quantify the NSS_FPG_.

#### 4.7.2. Micronucleus Test

The MN test was performed as previously reported [48]. Briefly, MN frequency in erythrocytes was evaluated through blood smears on glass slides. Two slides were prepared for each animal. The preparations were air-dried, fixed in methanol for 10 min, and stained in 5% Giemsa for 30 min. One thousand erythrocytes and the respective micronuclei were counted per slide (2000 cells for each animal) under an optical microscope (Nikon Eclipse E100). The MN frequencies were presented as mean ± SD for each experimental group.

### 4.8. Statistical Analysis

Relative organ weights were calculated as the ratio of the organ weight to the animal’s bodyweight. The data were analyzed using IBM SPSS software, version 25. The statistical approach used analysis of variance (ANOVA), followed a Bonferroni test. A Chi-squared test was performed for the histological lesions. Student’s *t* tests were performed for the comet and micronucleus assays. In all tests, we compared the HPV^−/−^ and the HPV^+/−^ groups fed with different diets (*P. umbilicalis*-supplemented animals and base diet-fed animals). So, group I compared to group III and group II to group IV. Differences were considered statistically significant in all the analyses when *p* < 0.05.

## 5. Conclusions

Red seaweed *P. umbilicalis* reduced the incidence of dysplastic cutaneous lesions induced by HPV16 in this model, suggesting that dietary supplementation with this seaweed in the concentration used may have beneficial chemopreventive effects. Results also indicate that the tested level of *P. umbilicalis* supplementation was safe and did not induce toxicity under the current experimental conditions.

## Figures and Tables

**Table 1:** Relative weight of organs in the experimental groups (mean ± standard error).

	Liver	Right Kidney	Left Kidney	Spleen	Lung	Heart	Bladder	Thymus
Group I (HPV16^−/−^, *Porphyra umbilicalis*)	0.0541 ± 0.0049	0.0060 ± 0.0002	0.0058 ± 0.0003	0.0040 ± 0.0002	0.0052 ± 0.0002 ^1^	0.0037 ± 0.0002	0.0006 ± 0.0001	0.0010 ± 0.0001
Group II (HPV16^+/−^, *P. umbilicalis*)	0.0670 ± 0.0015	0.0067 ± 0.0002	0.0065 ± 0.0001	0.0063 ± 0.0007	0.0063 ± 0.0003	0.0046 ± 0.0002	0.0010 ± 0.0001	0.0011 ± 0.0001
Group III (HPV16^−/−^, base diet)	0.0574 ± 0.0012	0.0057 ± 0.0002	0.0062 ± 0.0002	0.0047 ± 0.0002	0.0063 ± 0.0003	0.0042 ± 0.0002	0.0003 ± 0.0002	0.0012 ± 0.0002
Group IV (HPV16^+/−^, base diet)	0.0717 ± 0.0019	0.0069 ± 0.0002	0.0068 ± 0.0002	0.0083 ± 0.0010	0.0071 ± 0.0002	0.0051 ± 0.0002	0.0008 ± 0.0001	0.0014 ± 0.0001

^1^*p* = 0.016 statistically different from group III.

**Table 2:** Incidence of histological lesions in skin chest and ear samples in the experimental groups.

	Skin Chest Incidence/*n* (%)			Ear Incidence/*n* (%)		
	Normal	Epidermal Hyperplasia	Epidermal Dysplasia	Normal	Epidermal Hyperplasia	Epidermal Dysplasia
Group I (HPV16^−/−^, *P. umbilicalis*)	11/11 (100.0%)	0/11 (0%)	0/11 (0%)	11/11 (100.0%)	0/11 (0%)	0/11 (0%)
Group II (HPV16^+/−^, *P. umbilicalis*)	0/11 (0%)	11/11 (100.0%)	0/11 (0%)	0/11 (0%)	7/9 (77.8%)	2/9 (22.2%)
Group III (HPV16^−/−^, base diet)	11/11 (100.0%)	0/11 (0%)	0/11 (0%)	11/11 (100.0%)	0/11 (0%)	0/11 (0%)
Group IV (HPV16^+/−^, base diet)	0/11 (0%)	4/11 ^1^ (36.4%)	7/11 (63.6%)	0/11 (0%)	4/11 (36.4%)	7/11 (63.6%)

^1^*p* = 0.004, statistically different from group II.

**Table 3:** Serum biochemical parameters (mean ± standard error).

	Group I (HPV16^−/−^*, P. umbilicalis*)	Group II (HPV16^+/−^, *P. umbilicalis*)	Group III (HPV16^−/−^ Base Diet)	Group IV (HPV16^+/−^ Base Diet)
Albumin (g/L)	28.65 ± 1.37	30.43 ± 0.93	29.78 ± 1.71	30.37 ± 0.96
Total proteins (g/L)	45.95 ± 1.72	50.32 ± 2.24	51.34 ± 4.07	49.62 ± 1.12
Glucose (mg/dL)	222.29 ± 11.16	197.65 ± 15.97	195.70 ± 15.99	198.07 ± 13.36
Aspartate aminotransferase (U/L)	35.63 ± 4.03	40.89 ± 5.65	37.28 ± 4.70	38.85 ± 3.54
Alanine aminotransferase (U/L)	59.34 ± 5.17	65.66 ± 7.16	44.74 ± 3.76	51.82 ± 3.70
Gamma glutamyltransferase (U/L)	31.75 ± 3.17	40.07 ± 7.67	48.61 ± 6.11	60.78 ± 8.35
